# High interobserver variability of PTEN immunohistochemistry defining PTEN status in low- to intermediate-risk prostate cancer: results of the first German ring trial

**DOI:** 10.1007/s00428-024-03999-y

**Published:** 2024-12-09

**Authors:** Oliver Hommerding, Marit Bernhardt, Tobias Kreft, Anna Scherping, Mahmoud Abbas, Gustavo Baretton, Jan Hinrich Bräsen, Johannes Breyer, Christopher Darr, Franz Friedrich Dressler, Jörg Ellinger, Ramona Erber, Irene Esposito, Arndt Hartmann, Wolfgang Hartmann, Barbara Heitplatz, Hans Kreipe, Marcel Lafos, Johannes Linxweiler, Cristina Lopez-Cotarelo, Verena Sailer, Henning Reis, Matthias Saar, Hans-Ulrich Schildhaus, Katrin Schlack, Matthias Schmid, Maximilian Seidl, Axel Semjonow, Ulrich Sommer, Phillip Rolf Stahl, Verena Tischler, Florian Weber, Anna-Lena Wulf, Bernd Wullich, Glen Kristiansen

**Affiliations:** 1 Institute of Pathology, University Hospital Bonn, Bonn, Germany; 2 Department of Urology, University Hospital Bonn, Bonn, Germany; 3 Institute for Medical Biometry, Informatics and Epidemiology, University Hospital Bonn, Bonn, Germany; 4 Current Affiliation: Department of Urology, Aachen University (RWTH), Aachen, Germany; 5 Institute of Pathology, Heinrich Heine University and University Hospital of Duesseldorf, Dusseldorf, Germany; 6 Institute of Pathology, Comprehensive Cancer Center Erlangen-EMN, University Hospital Erlangen, Friedrich-Alexander-Universität Erlangen-Nürnberg (FAU), Erlangen, Germany; 7 Department of Urology, University Hospital Erlangen, Friedrich-Alexander-Universität Erlangen-Nürnberg (FAU), Erlangen, Germany; 8 Institute of Pathology, University Medicine Essen, University of Duisburg Essen, Essen, Germany; 9 Department of Urology, University Medicine Essen, University of Duisburg Essen, Essen, Germany; 10 Dr. Senckenberg Institute of Pathology, University Hospital Frankfurt, Goethe University Frankfurt, Frankfurt, Germany; 11 Department of Pathology, MSB Medical School Berlin, 14197 Berlin, Germany; 12 Department of Urology, Saarland University, Saarbrücken, Homburg/Saar Germany; 13 Institute of Pathology, University Hospital Muenster, Muenster, Germany; 14 Prostate Center, University Hospital Muenster, Muenster, Germany; 15 Institute of Pathology, University of Regensburg, Regensburg, Germany; 16 Department of Urology, Caritas Hospital St. Josef, University of Regensburg, Regensburg, Germany; 17 Institute of Pathology, Hannover Medical School, Hannover, Germany; 18 Institute of Pathology, University of Technology Dresden, Dresden, Germany; 19 Institute of Pathology, University Medical Center Schleswig-Holstein, Lübeck Site, Lübeck, Germany; 20 Institute of Pathology, Charité-Universitätsmedizin Berlin, Corporate Member of Freie Universität Berlin, Humboldt-Universität Zu Berlin, and Berlin Institute of Health, Berlin, Germany; 21 Discovery Life Sciences, Kassel, Germany; 22 Institute of Pathology, University Medical Center Mannheim, University of Heidelberg, Mannheim, Germany

**Keywords:** Prostate cancer, PTEN, Prognostic biomarker, Active surveillance, AKT

## Abstract

The prognostication of individual disease trajectory and selection of optimal therapy in patients with localized, low-grade prostate cancer often presents significant difficulty. The phosphatase and tensin homolog on chromosome 10 (PTEN) has emerged as a potential novel biomarker in this clinical context, based on its demonstrated prognostic significance in multiple retrospective studies. Incorporation into standard clinical practice necessitates exceptional diagnostic accuracy, and PTEN’s binary readout—retention or loss—suggests its suitability as a biomarker. This multi-institutional ring trial aimed to validate the diagnostic precision of PTEN immunohistochemistry in localized, low- to intermediate-risk prostate cancer, across ten university pathology institutes in Germany. The trial incorporated 90 cases of patients diagnosed with acinar adenocarcinoma of the prostate of grade groups 1 (*n* = 8, 8.9%) and 2 (*n* = 82, 91.1%) post-radical prostatectomy. Remarkably, the interpretation of PTEN immunohistochemistry displayed substantial variation (12.5–51.2% PTEN loss rates) within an identical cohort of prostate cancer. Fluorescence in situ hybridization analysis demonstrated *PTEN* hemizygous deletions in 5.5% (5/90) of cases. All cases with hemizygous deletions presented a distinct loss of PTEN expression by immunohistochemistry and were unanimously identified as PTEN loss by all participants (sensitivity 100%). However, negative (loss) immunohistochemistry was relatively non-specific for an underlying genomic deletion. Improved inter-observer agreement was observed in a subsequent ring trial. Finally, we identify S473-pAKT immunohistochemistry as a useful marker in equivocal cases. In summary, this multi-institutional ring trial illustrates surprisingly heterogeneous outcomes in defining PTEN status by immunohistochemistry.

**Supplementary Information:**

The online version contains supplementary material available at 10.1007/s00428-024-03999-y.

## Introduction

The differentiation of lethal, curable, and insignificant tumors, ideally at the time of diagnosis, is a prerequisite for individualized therapy in men with prostate cancer. While the indication for definitive therapy is clear in the majority of patients, there is often disagreement about whether active surveillance therapy should be considered in a subset of patients with low- to early intermediate-risk tumors. Clinicopathological criteria for evaluating active surveillance therapy include Gleason score, tumor volume in core needle biopsies, percentage of biopsy core involvement and serum PSA level. Recently, histological features like cribriform Gleason pattern 4 and intraductal carcinoma (IDC-P) were found to prognosticate aggressive disease and thus should exclude patients from active surveillance therapy [1–5]. A comprehensive understanding of molecular alterations in prostate cancer led to the development of novel molecular biomarkers for disease prognostication that might aid clinical decision-making in low- to intermediate-risk tumors. In addition to Ki-67 labeling index and RNA-based assays, PTEN (phosphatase and tensin homolog on chromosome 10), a negative regulator of the oncogenic PI3K-AKT-mTOR pathway, turned out as a promising novel biomarker in this setting [6].

Patients with tumors with PTEN loss are more likely to have biochemical recurrence (BCR) after radical prostatectomy compared to patients with PTEN intact tumors [20–23]. Detection of PTEN loss in core needle biopsies of grade group 1 and grade group 2 tumors increases the likelihood of upgrading, non-organ-confined disease (NOCD), and BCR after radical prostatectomy [24, 25]. However, due to a lack of prospective validation studies on active surveillance cohorts, PTEN status has not yet been implemented in standard clinical practice for the evaluation of active surveillance therapy [6].

Once implemented in clinical testing algorithms, it is reasonable to evaluate PTEN status primarily via immunohistochemistry because of its fast and seemingly straightforward interpretation with a dichotomous staining pattern, its lower costs compared to DNA-based tests, the usability on small biopsy specimen, and the implementation of PTEN immunohistochemistry in aforementioned studies. In addition, immunohistochemical analysis of PTEN detects protein loss not only caused by genomic deletion but also caused by alternative mechanisms like gene mutation, epigenetic silencing, and posttranscriptional and posttranslational regulation, that would be missed by *PTEN* FISH analysis. Current testing algorithms propose *PTEN* FISH or sequencing for equivocal immunohistochemical testing results [28].

High diagnostic accuracy is crucial for the integration of a biomarker into standard clinical practice. Ten university hospitals that participate in a project aiming to collect molecular data on prostate cancer prospectively (“SUMUS” trial) planned to start collecting PTEN data in primary prostate cancer cases. Before commencing the actual data accrual, the participants wanted to compare their established PTEN immunohistochemistry assays in order to assure future comparability of the data.

## Material and methods

### Case selection and construction of TMA

Ninety patients diagnosed with acinar adenocarcinoma of the prostate including grade groups 1 (8/90, 8.9%) and 2 (82/90, 91.1%) after radical prostatectomy at a single institute (Institute of Pathology, University Hospital Bonn, Bonn, Germany) were included in this study. Table 1 states the clinicopathological data of the cohort. None of the patients received neoadjuvant therapy. Formalin-fixed, paraffin-embedded tissue was retrieved from the archive to construct a tissue microarray (TMA) for a first ring trial consisting of one tumor spot (diameter 1 mm) from each of the 90 patients. PTEN loss rates of all participating institutes and the correlation of immunohistochemical results to genomic *PTEN* FISH data were supplied to all participants at the end of the first ring trial. For the second ring trial, a smaller TMA comprising 19 cases selected from the initial cohort was constructed, consisting of four unequivocal loss cases, five unequivocal retained cases, and 10 cases with equivocal rating. Table 2 states the clinicopathological data of the second cohort. For both the first and the second cohorts, tissue microarrays were cut in the lead institute (3-µm thick) and mounted on superfrost slides (Menzel Gläser, Brunswick, Germany). Two unstained slides were sent to all participating institutes and stained for PTEN according to local routine procedure. S473-pAKT, CD24, and GP2 immunohistochemistry was performed on randomly selected prostate needle core biopsies in which the PTEN status was determined by immunohistochemistry.

**Table 1:** Clinicopathological data of the cases included in the first ring trial

Clinical feature	Finding (*n* = 90)
Age, median (range), years	64 (49–76)
Preoperative PSA (ng/mL), median (range)	6.8 (1.4–54)
Postoperative parameters	
Grade Group at RP, *n* (%)	
1 (Gleason score 3 + 3 = 6), *n* (%)	8 (8.9%)
2 (Gleason score 3 + 4 = 7a), *n* (%)	82 (91.1%)
Organ-confined disease, *n* (%)	73 (81.1%)
Extraprostatic extension, *n* (%)	17 (18.9%)
Seminal vesicle involvement, *n* (%)	2 (2.2%)
Lymph node positive, *n* (%)	1 (1.1%)
Positive margins, *n* (%)	22 (24.4%)

**Table 2:** Clinicopathological data of the cases included in the second ring trial

Clinical feature	Finding (*n* = 19)
Age, median (range), years	65.0 (50–76)
Preoperative PSA (ng/mL), median (range)	8.0 (1.4–17.0)
Postoperative parameters	
Grade group at RP, *n* (%)	
1. *n* (%)	1 (5.3%)
2. *n* (%)	18 (94.7%)
Organ-confined disease, *n* (%)	16 (84.2%)
Extraprostatic extension, *n* (%)	3 (15.8%)
Seminal vesicle involvement, *n* (%)	0 (0%)
Lymph node positive, *n* (%)	1 (5.3%)
Positive margins, *n* (%)	4 (21.1%)

### Participants, immunohistochemistry protocols, and readout

Altogether, 10 institutes of pathology involved in the German multicentric Network Study of Molecular Alterations in urological Tumors (“SUMUS”) participated in this ring trial. The idea of the trial was to determine the *status quo* of PTEN diagnostics; hence—analogous to most commercially available ring trials in Europe—no specific antibody or protocol was recommended. Three out of 10 institutes used the primary anti-PTEN antibody clone D4.3 XP, a rabbit monoclonal antibody (Cell Signaling Technology, Catalogue number 9188). Five out of 10 institutes used the primary anti-PTEN antibody clone SP218, a rabbit monoclonal antibody (Abcam, catalog number ab228466). Two out of 10 institutes used the primary anti-PTEN antibody clone 138G6 rabbit monoclonal antibody (Cell Signaling Technology, catalog number 9559). One institute used a primary anti-PTEN rabbit antibody (Cell Signaling Technology, catalog number 9552). Two institutes stained both slides provided with different antibodies (clone SP218 and clone 138G6). The scoring options were determined as either loss of PTEN expression or retained PTEN expression. Following local staining and interpretation, the results were returned to the lead institute in Bonn. Thereafter, feedback was supplied to all participating institutes including a comparison between the participating institutes and the correlation of immunohistochemical results to *PTEN* FISH data.

Phospho-AKT immunohistochemistry was performed in the lead institute using an anti-AKT1 rabbit monoclonal antibody against phosphorylated at Serine 473 (1: 50, Abcam, ab81283, Cambridge, UK). The CD24 immunohistochemistry was performed as described with clone SWA11) [29]. GP2 immunohistochemistry was performed using an anti-GP2 rabbit monoclonal antibody (1: 400, Biozol Diagnostica, Eching, Germany).

### Definition of consensus

Cases rated as loss or retained by > 75% of all institutes were regarded as consensus voting [30].

### PTEN FISH analysis

Dual-color FISH was performed on the tissue microarray using the Vysis LSI PTEN/CEP 10 FISH Probe Kit according to the manufacturer’s instructions (Abbott Laboratories, Order-Nr. 04N62-020). Hemizygous deletion of *PTEN* was defined as > 20% of tumor nuclei containing a single *PTEN* locus signal. A Leica DM5500 B fluorescence microscope (Leica, Wetzlar, Germany) was used for FISH analysis.

### Statistical analysis and software

Statistical analysis was performed using the R software, version 4.1.0 (R Core Team (2021), R: A Language and Environment for Statistical Computing), R Foundation for Statistical Computing, Vienna, Austria. Inter-observer agreement was evaluated using Fleiss’ kappa with 95% confidence interval (CI). Fisher’s exact test was used for calculating statistical significance of S479-pAKT, CD24, and GP2 staining results. Graphs were generated with Prism, version 9.5.1, GraphPad Software, LLC.

## Results

### Analysis of PTEN expression in localized prostate cancer by immunohistochemistry shows low inter-observer agreement

Overall, 90 cases of patients diagnosed with acinar adenocarcinoma of the prostate of grade groups 1 (*n* = 8, 8.9%) and 2 (*n* = 82, 91.1%) after radical prostatectomy were included in this trial. A tissue microarray was constructed and provided to the 10 participating institutes. Two institutes evaluated staining results for two different anti-PTEN antibody clones resulting in 12 different data sets. Due to preprocessing artifacts (e.g., no tissue or no tumor on some TMA spots), of the 90 cases provided, the percentage of evaluable cases ranged from 66 to 88%. For 58 of the 90 cases (64.4%), all 12 datasets were entirely obtained.

**Fig. 1:**
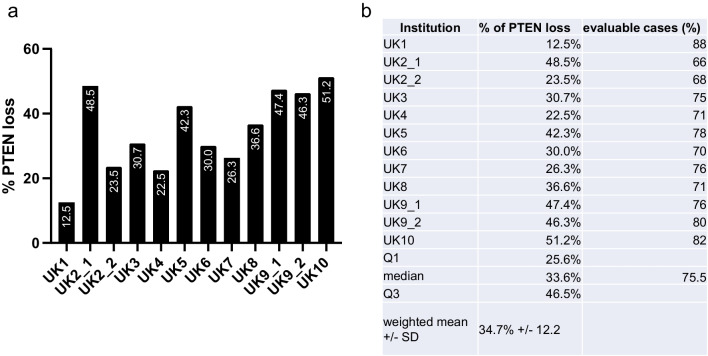
Results of the first ring trial. **a** Graphical and **b** tabular overview of PTEN immunohistochemistry results among the institutes in the first ring trial

**Fig. 2:**
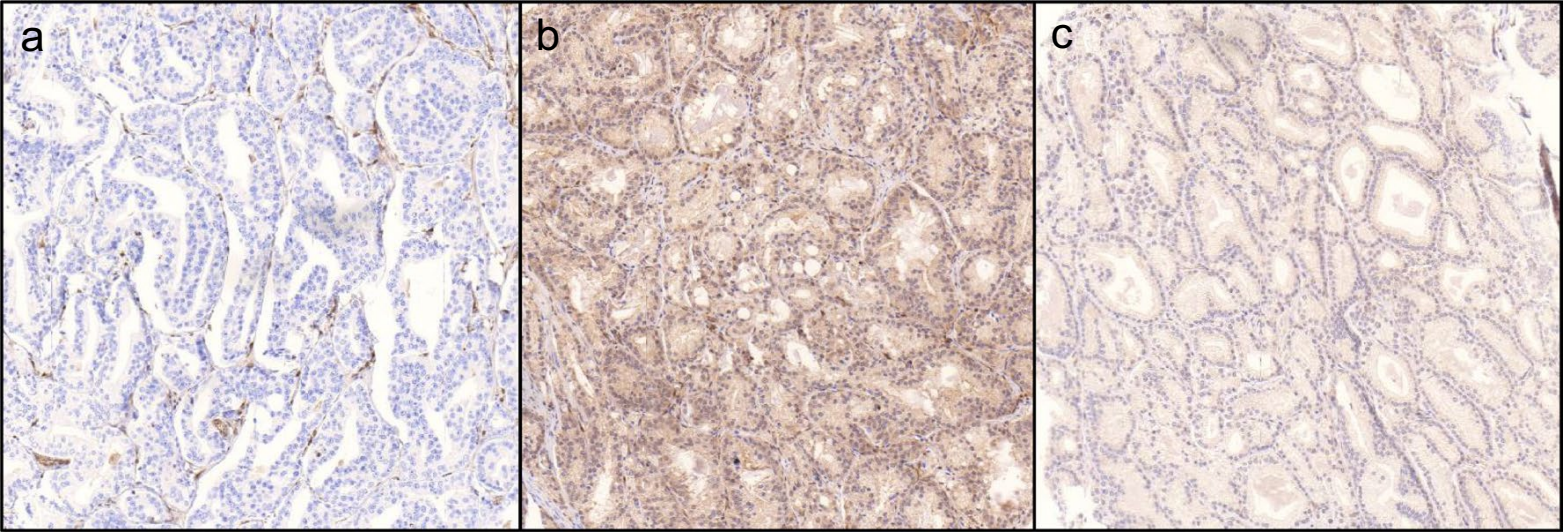
Representative PTEN immunohistochemistry. **a** Homogenous loss of PTEN expression in tumor glands with intact staining in peritumoral stroma. These cases was rated ‘PTEN loss’ by all institutes. **b** Homogenous and strong PTEN expression in tumor glands and stroma. This case was rated ‘retained PTEN expression’ by all institutes. **c** Equivocal case with weak PTEN expression. This case was rated ‘PTEN loss’ by 5 institutes and ‘retained PTEN expression’ by 5 institutes

Reported PTEN-loss rates among participating institutes ranged from 12.5 to 51.2% (mean 34.7% + / − 12.2 SD) (Fig. 1). Cases classified as loss or retained by > 75% of all institutes were regarded as consensus voting [30]. Using this definition of a consensus rating in total, 72% of cases (65/90 cases) showed a consensus rating for either PTEN loss or retained PTEN expression while an equivocal rating was found in 28% of the cases (25/90 cases). Consensus rating was preferentially found in cases showing either strong PTEN expression or total loss of PTEN expression (Fig. 2a, b). Equivocal cases showing low inter-observer agreement had particularly weak or doubtful PTEN expression (Fig. 2c). Fleiss’ kappa coefficient for the 58 datasets (64.4%), that were entirely obtained, was 0.54, 95% CI = [0.50; 0.57].

### FISH analysis

**Fig. 3:**
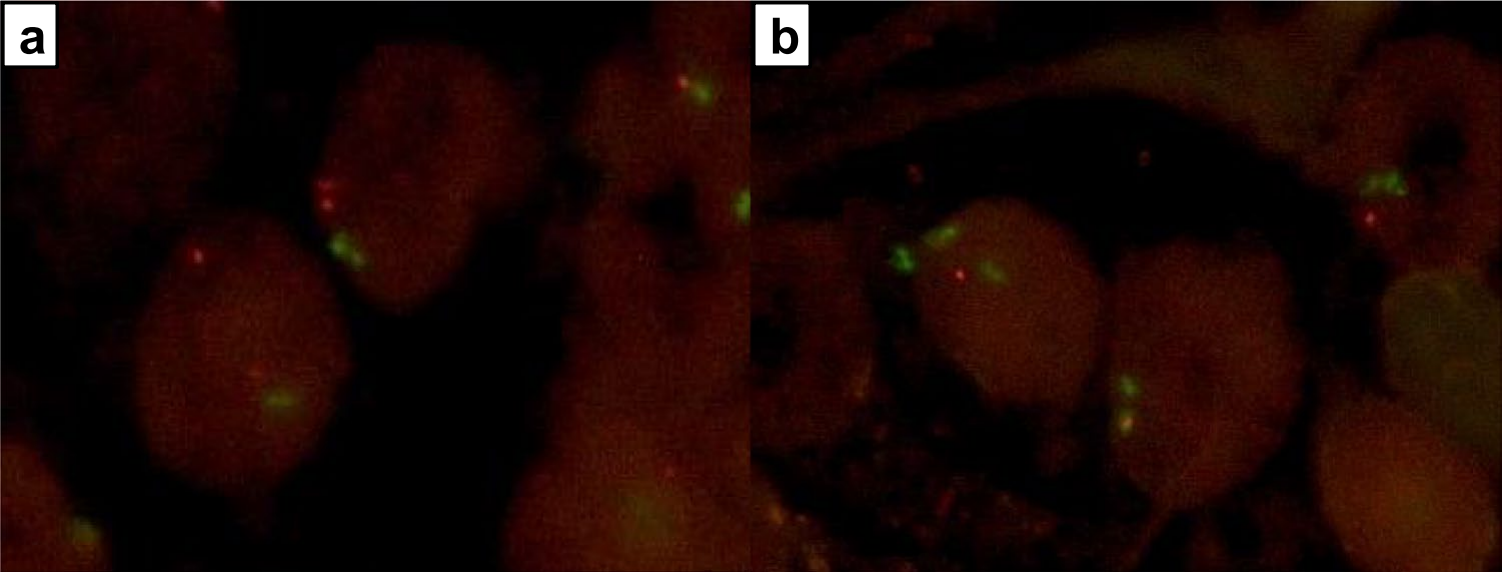
PTEN FISH analysis. The LSI PTEN probe hybridizes to the 10q23 region on chromosome 10 (red signal). The CEP 10 SpectrumGreen probe hybridizes to chromosome 10 (green signal). **a** Tumor sample with retained PTEN expression and two intact PTEN alleles. **b** Tumor sample with PTEN loss and hemizygous PTEN deletion (red)

To compare the immunohistochemical results with the genomic PTEN status, all 90 cases were analyzed for *PTEN* deletions by FISH analysis. *PTEN* hemizygous deletions were found in 5.6% of cases (5/90) whereas no homozygous deletions could be detected (Fig. 3). All five cases with hemizygous deletions showed a complete loss of PTEN expression by immunohistochemistry and were designated as PTEN loss by all participants. Therefore, a positive (retained pattern) immunohistochemistry result ruled out genomic PTEN loss with a sensitivity of 100%. However, a negative (loss pattern) immunohistochemistry result was rather unspecific for an underlying genomic deletion; the latter being found in 11.9 to 45.5% of all cases with PTEN loss. Thus, the specificity of immunohistochemistry to detect a genomic deletion thus ranged from 52.0 to 92.8% (Table 1).

### Repeated analysis of PTEN expression improves inter-observer agreement

**Fig. 4:**
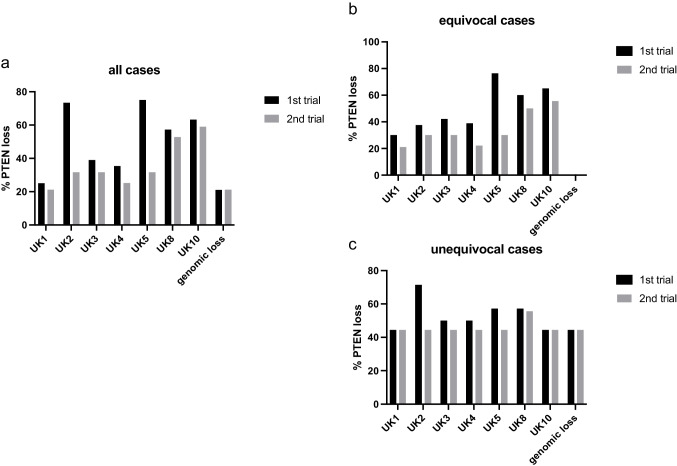
Results of the second ring trial in comparison to the first trial. Results of **a** all cases, **b** equivocal cases only and **c** and unequivocal cases only

As the rather poor inter-observer agreement seems unacceptable for clinical use, we aimed to test for a possibly improved inter-observer agreement in a subsequent second trial following feedback to the participants. Therefore, PTEN loss rates of all participating institutes and the correlation of immunohistochemical results to genomic *PTEN* FISH data were supplied to all participants. Next, a smaller tissue microarray comprising 19 cases selected from the cohort was constructed, consisting of four unequivocal loss cases, five unequivocal retained cases, and 10 cases with equivocal ratings. In all four loss cases (4/19, 21%), a hemizygous *PTEN* deletion was identified by FISH analysis. Again, two unstained slides were sent to all institutes for repeated evaluation and seven out of 10 institutes participated in the second ring trial. Similar to the first ring trial, all four cases with hemizygous deletions were designated as PTEN loss by all participants. Therefore, a positive (retained pattern) immunohistochemistry signal again ruled out genomic PTEN loss (sensitivity 100%). As expected, the mean of the PTEN loss rate was different due to the different composition of the second TMA (Fig. 4a). However, a marked harmonization of the inter-observer agreement was noted especially in equivocal cases (Fig. 4b, c). Fleiss’ kappa coefficient for the 16 datasets (82.4%) of the second ring trial that were entirely obtained increased to 0.64, 95% CI = [0.53; 0.75], compared to 0.54, 95% CI = [0.50; 0.57], in the first ring trial. When calculating the Fleiss’ kappa coefficient for the datasets of the first ring trial of only the participants of the second trial, the increase in Fleiss’ kappa coefficient was even more pronounced (0.40 vs. 0.64).

### Tentative ancillary surrogate markers of PTEN inactivation/loss

Despite the improvement in diagnostic accuracy for detecting PTEN loss through a repeated ring trial, individual cases still present diagnostic challenges. To address this, we investigated additional potential immunohistochemical markers that aid in the detection of PTEN loss in equivocal cases.

**Fig. 5:**
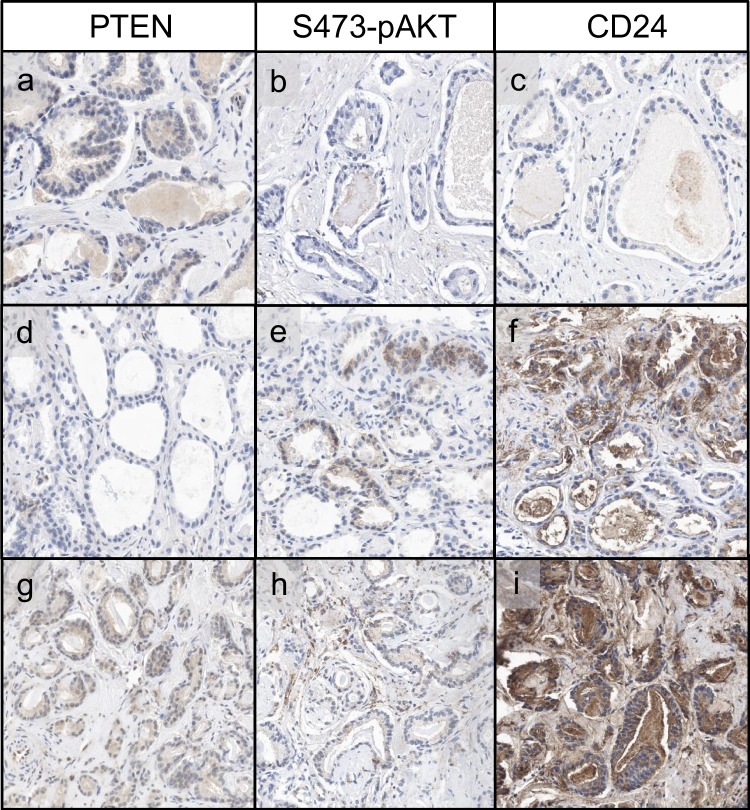
Expression of PTEN, S473-pAKT, and CD24 in prostate needle core biopses. **a**–**c** Prostate cancer with retained PTEN expression, absent S473-pAKT, and no expression of CD24. **d**–**f** Prostate cancer with loss of PTEN expression, positivity for S473-pAKT, and expression of CD24. **g**–**i** Prostate cancer with retained PTEN expression, weak equivocal staining for S473-pAKT and diffuse overexpression of CD24

PTEN acts as a tumor suppressor by dephosphorylating PIP3 and antagonizing the PI3K-AKT pathway. PTEN loss results in the activation of PI3K, which subsequently leads to AKT activation via phosphorylation at two sites, T308 and S473, with maximal activation at S473 [31, 32]. We conducted immunohistochemical analysis of S473-pAKT in a total of 20 core biopsies of prostate cancer with both PTEN loss and retention. As anticipated, S473-pAKT was present in the majority of cancers with PTEN loss (7/9), whereas most cancers with retained PTEN expression showed no S473-pAKT expression (10/11) (Fig. 5a, b, d, e). However, few false negative and false positive results were noted (Fig. 5g, h). The sensitivity of S473-pAKT immunohistochemistry to detect PTEN loss was 77.8% with a high specificity of 90.9% (Table 3). Notably, the sensitivity was markedly decreased when tested on a tissue microarray of prostate cancer cases taken from prostatectomy specimens (16.3%). We attribute this to prolonged cold ischemia time in the center of the RPE specimen, as immediate fixation post-collection is essential for preservation of protein phosphorylation as shown in other tumors [33]. In concordance with this notion, preliminary results show a staining gradient for S473-pAKT in prostatectomy specimens, which we attribute to delayed fixation in the specimen center (Fig. S1). This gradient underscores the importance of immediate fixation post-collection, as previously noted for protein phosphorylation in other contexts.

**Table 3:** Staining results of PTEN, S473-pAKT, CD24, and GP2 on prostate needle core biopsies (*p*-values: S473-pAKT 0.0045, CD24 n.s., GP2 n.s.; Fisher’s exact test)

Marker	PTEN loss	PTEN retained
S479-AKT +	7	1
S479-AKT −	2	10
CD24 +	7	5
CD24 −	5	3
GP2 +	0	0
GP2 −	9	11

Additionally, we assessed CD24 expression as a potential immunohistochemical surrogate marker for PTEN loss, which we and others had found upregulated in cases with PTEN loss [7, 34]. We performed CD24 immunohistochemistry in the same core biopsies of prostate cancer with both PTEN loss and retention, which were already analyzed for S473-pAKT. CD24 expression was rather heterogenous compared to S473-pAKT expression and did not correlate with PTEN expression (Fig. 5c, f, i). The sensitivity of CD24 immunohistochemistry to detect PTEN loss was 58.3% with a rather low specificity of 37.5% (Table 3).

Thirdly, we picked GP2, an immune-associated gene, that also has been shown to be transcriptionally upregulated in prostate cancer with loss of PTEN expression in a prior screening study [35]. However, only focal expression of GP2 was found in one case of PTEN loss (Table 3). Thus, GP2 expression does not qualify as an immunohistochemical surrogate for PTEN loss.

## Discussion

Determining the aggressiveness of a tumor or predicting the course of localized low-grade prostate cancer is a challenging task. This is because there is a range of treatment options available, from immediate definitive therapy to active surveillance. To facilitate treatment decisions, molecular biomarkers in addition to established serological, radiological, and histological parameters are desired. At the ISUP consensus conference on molecular biomarkers in genitourinary tumors in 2019, Ki-67 and PTEN were identified as the most promising markers [6]{Lotan, 2020 #76}.

However, the application of Ki-67 as a biomarker is complicated by interobserver variability due to the reflective nature of the proliferative activity, which requires a cut-off value to determine prognostic subgroups [36–38]. On the other hand, PTEN is one of the most frequently altered genes in prostate cancer, and its prognostic and potential therapeutic implications have been demonstrated in multiple retrospective studies [20–25, 39]. PTEN appears to be a more reliable biomarker than Ki-67 because its readout is dichotomous, meaning it is either retained or lost. Immunohistochemistry is the preferred modality for assessing PTEN status due to its easy interpretation, inexpensiveness, and wide implementation in pathology laboratories.

PTEN loss in prostate cancer is mostly due to *PTEN* biallelic genomic deletion whereas *PTEN* mutations occur less frequently and in association with hemizygous deletions [7–13]. Gene rearrangement comprising the *PTEN* locus is reported as an additional mechanism of PTEN inactivation [14]. PTEN loss or downregulation via promotor hypermethylation, miRNAs or other mechanisms is less well studied [15, 16]. Due to an association with Gleason score and tumor stage, the reported prevalence of PTEN loss in prostate cancer is variable across different cohorts and is reported in about 20% of primary and in about 40% of metastatic tumors [7, 8, 10, 11, 17–19].

Apart from its role as a prognostic biomarker, it is expectable that PTEN status might have a role as a predictive biomarker in PTEN-deficient advanced prostate cancer. Promising treatment options focus on the blockage of the PI3K-AKT-mTOR pathway in combination with established therapeutics [26]. An ongoing phase III randomized double-blind study (IPATential150) testing the AKT-inhibitor ipatasertib with abiraterone and prednisone compared with placebo plus abiraterone and prednisone in patients with metastatic castrate-resistant prostate cancer with PTEN loss showed an improved radiographic progression-free survival in ipatasertib-treated patients [27].

In preparation of a prospective multicentric collection of molecular prostate cancer data including PTEN immunohistochemistry, a ring trial was conducted to determine the status quo of the diagnostic accuracy of PTEN immunohistochemistry in localized low- to intermediate-risk prostate cancer among ten eminent university pathology institutes in Germany. Unexpectedly, the interpretation of PTEN immunohistochemistry in this study showed marked variation (12.5–51.2% PTEN loss rates) in an identical cohort of prostate cancer. This may not be surprising, as no harmonization of the PTEN assay was conducted prior to this ring trial. While there was a trend towards harmonization in the interobserver agreement in a subsequent smaller ring trial following feedback to the participants, even these results require further improvement; hence, we think this data argues against the uncritical use of PTEN immunohistochemistry to define PTEN status for clinical routine use. So far, the interobserver variability of PTEN immunohistochemistry has been studied in only a few other entities. Because of the frequent *PTEN* loss in endometrial cancer, PTEN immunohistochemistry is well implemented as a routine diagnostic biomarker in this entity. Comparing the interobserver variability of *PTEN* loss in endometrial cancer between two institutes, it was found to be highly reproducible with a kappa value of 0.8 [40].

FISH analysis of our cohort revealed *PTEN* hemizygous deletions in 5.5% (5/90) of all cases, which is in contrast to the reported prevalence of *PTEN* loss in about 20% of primary prostate cancers [7, 8, 10]. However, this finding is likely to be explained by the selection of only grade group 1 and 2 tumors [20, 23, 41].

In direct comparison of immunohistochemical results with FISH analysis, the positive predictive value of the genomic alteration ranged from 11.9 to 45.5%. All cases with a hemizygous *PTEN* deletion were totally negative on immunohistochemistry, and conversely, any positive PTEN immunoreactivity ruled out a genomic *PTEN* loss (sensitivity 100%). However, negative (loss) immunohistochemistry was rather unspecific for an underlying genomic deletion. Then, 6.7% (6/90) of the tumors with a loss immunophenotype showed no genomic loss in the FISH analysis, and therefore, alternative mechanisms of *PTEN* inactivation such as gene rearrangement, gene mutation, epigenetic silencing, and posttranscriptional or posttranslational regulation are likely present in these cases. It remains elusive whether FISH analysis should, as proposed, be defined as the gold standard in equivocal immunohistochemistry cases, because *PTEN* loss by other mechanisms than genomic loss could be missed.

To enhance the diagnostic accuracy of determining PTEN status by immunohistochemistry, we explored potential surrogate markers for PTEN loss in prostate cancer. We focused on the activation of the PI3K-AKT pathway using S473-pAKT immunohistochemistry, which is indicative of PTEN loss [31, 32]. In a cohort of 20 prostate cancer cases, we found that S473-pAKT demonstrated a sensitivity of 77.8% and specificity of 90.9% for detecting PTEN loss. Consequently, S473-pAKT may serve as a surrogate marker for PTEN loss in cases where PTEN immunohistochemistry results are equivocal. Importantly, the sensitivity was markedly decreased when tested on samples from radical prostatectomy specimen, because being a phosphoprotein, S473-pAKT is an unstable protein modification that is sensitive to prolonged cold ischemia time. Therefore, short times to fixation is of paramount importance in the correct evaluation of S473-pAKT. In addition, we tested CD24 and GP2 expressions as additional immunohistochemical surrogate markers for PTEN loss. However, CD24 expression was heterogenous and showed a rather low specificity compared to S473-pAKT immunohistochemistry. GP2 expression was only found focally in one case of PTEN loss. Thus, S473-pAKT but not CD24 or GP2 may serve as an additional marker in cases where PTEN immunohistochemistry results are unclear.

Strengths of this study are its participants that reflect a representative fraction of academic pathology institutes in Germany and the deliberate selection of low-grade prostate cancer cases—exactly the group in which prognostic biomarkers are most urgently needed. Weaknesses of this study are its small to medium cohort size (*n* = 90), the use of a tissue microarray which limits the amount of tumor that can be studied, and the lack of follow-up data.

In summary, this ring trial investigates the variability of PTEN immunohistochemistry in low- to intermediate-risk prostate cancer. The data recommends that every institute should establish a PTEN immunohistochemistry protocol in close correlation with PTEN genomics and genetics. It also indicates that only a complete loss of immunoreactivity is diagnostic of a PTEN loss, as even the faintest positivity, that may be perceived as background, is indicative of a retained PTEN status. In addition, in equivocal cases, S473-pAKT immunohistochemistry may serve as an ancillary surrogate marker for PTEN loss. Regular interlaboratory ring trials are necessary to further improve the reliability and reproducibility of the PTEN assay.

## Supplementary Information

Below is the link to the electronic supplementary material.

Supplementary file1 Section from a basal margin of a radical prostectomy specimen showing cancer with PTEN loss (a). S473-pAKT staining shows a staining gradient with strong positivity at the specimen surface (lower image border) and reduced positivity in the center (b) (DOCX 741 KB)

## Data Availability

Data is available upon reasonable request.

## References

[1] Epstein JI, Amin MB, Fine SW et al (2021) The 2019 Genitourinary Pathology Society (GUPS) white paper on contemporary grading of prostate cancer. Arch Pathol Lab Med 145:461–493. 10.5858/ARPA.2020-0015-RA 32589068 10.5858/arpa.2020-0015-RA

[2] Hollemans E, Verhoef EI, Bangma CH et al (2021) Cribriform architecture in radical prostatectomies predicts oncological outcome in Gleason score 8 prostate cancer patients. Mod Pathol 34:184–193. 10.1038/S41379-020-0625-X 32686748 10.1038/s41379-020-0625-x PMC7806505

[3] Kato M, Tsuzuki T, Kimura K et al (2016) The presence of intraductal carcinoma of the prostate in needle biopsy is a significant prognostic factor for prostate cancer patients with distant metastasis at initial presentation. Mod Pathol 29:166–173. 10.1038/MODPATHOL.2015.146 26743470 10.1038/modpathol.2015.146

[4] Kweldam CF, Kümmerlin IP, Nieboer D et al (2016) Disease-specific survival of patients with invasive cribriform and intraductal prostate cancer at diagnostic biopsy. Mod Pathol 29:630–636. 10.1038/MODPATHOL.2016.49 26939875 10.1038/modpathol.2016.49

[5] van Leenders GJLH, Kweldam CF, Hollemans E et al (2020) Improved prostate cancer biopsy grading by incorporation of invasive cribriform and intraductal carcinoma in the 2014 grade groups. Eur Urol 77:191–198. 10.1016/J.EURURO.2019.07.051 31439369 10.1016/j.eururo.2019.07.051

[6] Lotan TL, Tomlins SA, Bismar TA et al (2020) Report from the International Society of Urological Pathology (ISUP) consultation conference on molecular pathology of urogenital cancers. I. Molecular biomarkers in prostate cancer. Am J Surg Pathol 44:E15–E29. 10.1097/PAS.0000000000001450 32044806 10.1097/PAS.0000000000001450

[7] Abeshouse A, Ahn J, Akbani R et al (2015) The molecular taxonomy of primary prostate cancer. Cell 163:1011–1025. 10.1016/J.CELL.2015.10.025 26544944 10.1016/j.cell.2015.10.025 PMC4695400

[8] Barbieri CE, Baca SC, Lawrence MS et al (2012) Exome sequencing identifies recurrent SPOP, FOXA1 and MED12 mutations in prostate cancer. Nat Genet 44:685–689. 10.1038/NG.2279 22610119 10.1038/ng.2279 PMC3673022

[9] Beltran H, Yelensky R, Frampton GM et al (2013) Targeted next-generation sequencing of advanced prostate cancer identifies potential therapeutic targets and disease heterogeneity. Eur Urol 63:920–926. 10.1016/J.EURURO.2012.08.053 22981675 10.1016/j.eururo.2012.08.053 PMC3615043

[10] Berger MF, Lawrence MS, Demichelis F et al (2011) The genomic complexity of primary human prostate cancer. Nature 470:214–220. 10.1038/NATURE09744 21307934 10.1038/nature09744 PMC3075885

[11] Grasso CS, Wu YM, Robinson DR et al (2012) The mutational landscape of lethal castration-resistant prostate cancer. Nature 487:239–243. 10.1038/NATURE11125 22722839 10.1038/nature11125 PMC3396711

[12] Robinson D, Van Allen EM, Wu YM et al (2015) Integrative clinical genomics of advanced prostate cancer. Cell 161:1215–1228. 10.1016/J.CELL.2015.05.001 26000489 10.1016/j.cell.2015.05.001 PMC4484602

[13] Phin S, Moore MW, Cotter PD (2013) Genomic rearrangements of PTEN in prostate cancer. Front Oncol 3. 10.3389/FONC.2013.00240 10.3389/fonc.2013.00240 PMC3775430 24062990

[14] Reid AHM, Attard G, Brewer D et al (2012) Novel, gross chromosomal alterations involving PTEN cooperate with allelic loss in prostate cancer. Mod Pathol 25:902–910. 10.1038/MODPATHOL.2011.207 22460813 10.1038/modpathol.2011.207

[15] Konishi N, Nakamura M, Kishi M et al (2002) Heterogeneous methylation and deletion patterns of the INK4a/ARF locus within prostate carcinomas. Am J Pathol 160:1207–1214. 10.1016/S0002-9440(10)62547-3 11943705 10.1016/S0002-9440(10)62547-3 PMC1867197

[16] Whang YE, Wu X, Suzuki H et al (1998) Inactivation of the tumor suppressor PTEN/MMAC1 in advanced human prostate cancer through loss of expression. Proc Natl Acad Sci U S A 95:5246–5250. 10.1073/PNAS.95.9.5246 9560261 10.1073/pnas.95.9.5246 PMC20246

[17] Han B, Mehra R, Lonigro RJ et al (2009) Fluorescence in situ hybridization study shows association of PTEN deletion with ERG rearrangement during prostate cancer progression. Mod Pathol 22:1083–1093. 10.1038/MODPATHOL.2009.69 19407851 10.1038/modpathol.2009.69 PMC2760294

[18] Liu W, Laitinen S, Khan S et al (2009) Copy number analysis indicates monoclonal origin of lethal metastatic prostate cancer. Nat Med 15:559–565. 10.1038/NM.1944 19363497 10.1038/nm.1944 PMC2839160

[19] Taylor BS, Schultz N, Hieronymus H et al (2010) Integrative genomic profiling of human prostate cancer. Cancer Cell 18:11–22. 10.1016/J.CCR.2010.05.026 20579941 10.1016/j.ccr.2010.05.026 PMC3198787

[20] Krohn A, Diedler T, Burkhardt L et al (2012) Genomic deletion of PTEN is associated with tumor progression and early PSA recurrence in ERG fusion-positive and fusion-negative prostate cancer. Am J Pathol 181:401–412. 10.1016/j.ajpath.2012.04.026 22705054 10.1016/j.ajpath.2012.04.026

[21] Lotan TL, Wei W, Morais CL et al (2016) PTEN loss as determined by clinical-grade immunohistochemistry assay is associated with worse recurrence-free survival in prostate cancer. Eur Urol Focus 2:180–188. 10.1016/J.EUF.2015.07.005 27617307 10.1016/j.euf.2015.07.005 PMC5014432

[22] Mehra R, Salami SS, Lonigro R et al (2018) Association of ERG/PTEN status with biochemical recurrence after radical prostatectomy for clinically localized prostate cancer. Med Oncol 35. 10.1007/S12032-018-1212-6 10.1007/s12032-018-1212-6 PMC6601345 30291535

[23] Yoshimoto M, Cunha IW, Coudry RA et al (2007) FISH analysis of 107 prostate cancers shows that PTEN genomic deletion is associated with poor clinical outcome. Br J Cancer 97:678–685. 10.1038/SJ.BJC.6603924 17700571 10.1038/sj.bjc.6603924 PMC2360375

[24] Guedes LB, Tosoian JJ, Hicks J et al (2017) PTEN loss in Gleason Score 3 + 4 = 7 prostate biopsies is associated with nonorgan confined disease at radical prostatectomy. J Urol 197:1054–1059. 10.1016/J.JURO.2016.09.084 27693448 10.1016/j.juro.2016.09.084

[25] Lotan TL, Carvalho FL, Peskoe SB et al (2015) PTEN loss is associated with upgrading of prostate cancer from biopsy to radical prostatectomy. Mod Pathol 28:128–137. 10.1038/MODPATHOL.2014.85 24993522 10.1038/modpathol.2014.85 PMC4282985

[26] Turnham DJ, Bullock N, Dass MS et al (2020) The PTEN conundrum: how to target PTEN-deficient prostate cancer. Cells 9. 10.3390/CELLS9112342 10.3390/cells9112342 PMC7690430 33105713

[27] Sweeney C, Bracarda S, Sternberg CN et al (2021) Ipatasertib plus abiraterone and prednisolone in metastatic castration-resistant prostate cancer (IPATential150): a multicentre, randomised, double-blind, phase 3 trial. Lancet 398:131–142. 10.1016/S0140-6736(21)00580-8 34246347 10.1016/S0140-6736(21)00580-8

[28] Jamaspishvili T, Berman DM, Ross AE et al (2018) Clinical implications of PTEN loss in prostate cancer. Nat Rev Urol 15:222–234. 10.1038/NRUROL.2018.9 29460925 10.1038/nrurol.2018.9 PMC7472658

[29] Kristiansen G, MacHado E, Bretz N et al (2010) Molecular and clinical dissection of CD24 antibody specificity by a comprehensive comparative analysis. Lab Invest 90:1102–1116. 10.1038/labinvest.2010.70 20351695 10.1038/labinvest.2010.70

[30] Diamond IR, Grant RC, Feldman BM et al (2014) Defining consensus: a systematic review recommends methodologic criteria for reporting of Delphi studies. J Clin Epidemiol 67:401–409. 10.1016/J.JCLINEPI.2013.12.002 24581294 10.1016/j.jclinepi.2013.12.002

[31] Alessi DR, Andjelkovic M, Caudwell B et al (1996) Mechanism of activation of protein kinase B by insulin and IGF-1: insulin signalling/phosphatidylinositol 3-kinase/protein kinase B/protein phosphorylation. EMBO J 15(23):6541-51 PMC452479 8978681

[32] Maehama T, Dixon JE (1998) The tumor suppressor, PTEN/ MMAC1, dephosphorylates the lipid second messenger, phosphatidylinositol 3,4,5-trisphosphate. J Biol Chem 273(22):13375-8 10.1074/jbc.273.22.13375 9593664

[33] Beccari S, Mohamed E, Voong V et al (2024) Quantitative assessment of preanalytic variables on clinical evaluation of PI3/AKT/mTOR signaling activity in diffuse glioma. Modern Pathology 37. 10.1016/j.modpat.2024.100488 10.1016/j.modpat.2024.100488 38588881

[34] Tolkach Y, Zarbl R, Bauer S et al (2021) DNA promoter methylation and ERG regulate the expression of CD24 in prostate cancer. Am J Pathol 191:618–630. 10.1016/j.ajpath.2020.12.014 33485866 10.1016/j.ajpath.2020.12.014

[35] Imada EL, Sanchez DF, Dinalankara W et al (2021) Transcriptional landscape of PTEN loss in primary prostate cancer. BMC Cancer 21. 10.1186/s12885-021-08593-y 10.1186/s12885-021-08593-y PMC8314517 34311724

[36] Laenkholm AV, Grabau D, MøllerTalman ML et al (2018) An inter-observer Ki67 reproducibility study applying two different assessment methods: on behalf of the Danish Scientific Committee of Pathology, Danish breast cancer cooperative group (DBCG). Acta Oncol 57:83–89. 10.1080/0284186X.2017.1404127 29202622 10.1080/0284186X.2017.1404127

[37] Polley MYC, Leung SCY, McShane LM et al (2013) An international Ki67 reproducibility study. J Natl Cancer Inst 105:1897–1906. 10.1093/JNCI/DJT306 24203987 10.1093/jnci/djt306 PMC3888090

[38] Raap M, Ließem S, Rüschoff J et al (2017) Quality assurance trials for Ki67 assessment in pathology. Virchows Arch 471:501–508. 10.1007/S00428-017-2142-Y 28497316 10.1007/s00428-017-2142-y

[39] Ahearn TU, Pettersson A, Ebot EM et al (2016) A prospective investigation of PTEN loss and ERG expression in lethal prostate cancer. J Natl Cancer Inst 108. 10.1093/jnci/djv346 10.1093/jnci/djv346 PMC4862436 26615022

[40] Garg K, Broaddus RR, Soslow RA et al (2012) Pathologic scoring of PTEN immunohistochemistry in endometrial carcinoma is highly reproducible. Int J Gynecol Pathol 31:48–56. 10.1097/PGP.0B013E3182230D00 22123723 10.1097/PGP.0b013e3182230d00 PMC4244710

[41] Troyer DA, Jamaspishvili T, Wei W et al (2015) A multicenter study shows PTEN deletion is strongly associated with seminal vesicle involvement and extracapsular extension in localized prostate cancer. Prostate 75:1206–1215. 10.1002/PROS.23003 25939393 10.1002/pros.23003 PMC4475421

