# Supplementary figures and images for: High interobserver variability of PTEN immunohistochemistry defining PTEN status in low- to intermediate-risk prostate cancer: results of the first German ring trial
Source: Virchows Arch. 2024 Dec 9;487(1):87–96. doi: 10.1007/s00428-024-03999-y (PMC12289804; doi:10.1007/s00428-024-03999-y)

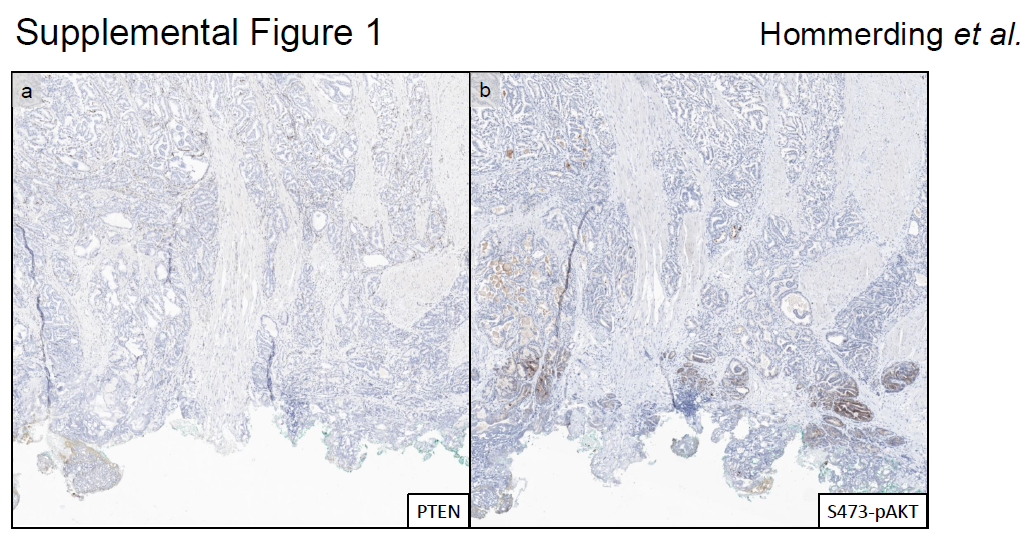

Supplement: Supplementary file 1 — Supplementary file1 Section from a basal margin of a radical prostectomy specimen showing cancer with PTEN loss (a). S473-pAKT staining shows a staining gradient with strong positivity at the specimen surface (lower image border) and reduced positivity in the center (b) (DOCX 741 KB) [file 428_2024_3999_MOESM1_ESM.docx]
